# Human olfactory sensitivity varies across geographical locations

**DOI:** 10.1038/s41598-026-38727-w

**Published:** 2026-02-18

**Authors:** Aleksandra Reichert, Nixon M. Abraham, Jancy N. Abraham, Maria Laura Albanese, Rafieh Alizadeh, Ines Aloulou, Lixin Chen, Ma. Lourdes Berioso Enecilla, Marco Aurélio Fornazieri, Johannes Frasnelli, Juan Martin Fuselli, Fatima Gansatao, Cagdas Guducu, Anna Kristina Hernandez, Marlise K. Hofer, Salina Husain, Reda Kamel, Elliott Lamond, Francesco Loy, Mehmet K. Mahmut, Daniel Marek, Carla Masala, Élizabeth Michaluk, Imen Miri, Marjan Mirsalehi, Plamena Miteva, Isabel Bernardes Moura, Anasuha Musa, Hanène Naija, Keigo Nakaachi, Jayant M. Pinto, Patricia Portillo Mazal, Ahmed Radwan, Farhad Rafiei, Devesh Rawat, Katarzyna Resler, Henrique O. Scussiatto, Hozifa Alsaid Sheta, Sharanya M. Thodupunoori, Brianna J. Turner, Nora Van Oosterhout, Hangying Wu, Ayaho Yoshino, Laiquan Zou, Barbara Zyzelewicz, Thomas Hummel, Anna Oleszkiewicz

**Affiliations:** 1 Institute of Psychology, University of Wroclaw, Wrocław, Poland; 2 Laboratory of Neural Circuits and Behaviour (LNCB), Department of Biology, Indian Institute of Science Education and Research (IISER), Pune, Maharashtra India; 3 Department of Life Sciences, Centre of Excellence in Epigenetics, Shiv Nadar Institution of Eminence, Uttar Pradesh, Gautam Buddha Nagar, Greater Noida, India; 4 Pontifícia Universidade Católica Do Paraná (PUC-PR), Paraná, Brazil; 5 ENT and Head and Neck Research Center and Department, School of Medicine, The Five Senses Health Institute, Iran University of Medical Sciences, Tehran, Iran; 6 Department of Physical and Rehabilitation Medicine, Research laboratory “Clinical Psychology: Intersubjectivity and Culture”, Institut Kassab, Manouba, Tunisia; 7 Chemical Senses and Mental Health Laboratory, Department of Psychology, School of Public Health, Southern Medical University, Guangzhou, Guangdong China; 8 St. Luke’s Medical Center, Quezon City, Manila, Philippines; 9 Universidade Estadual de Londrina (UEL), Londrina, Brazil; 10 Department of Anatomy, Université du Québec À Trois-Rivières, Trois-Rivières, Canada; 11 Hospital Naval Dr. Pedro Mallo, Buenos Aires, Argentina; 12 East Avenue Medical Center, Manila, Philippines; 13 Faculty of Medicine, Department of Biophysics, Dokuz Eylul University, Izmir, Turkey; 14 Department of Otorhinolaryngology, Faculty of Medicine Carl Gustav Carus, Smell & Taste Clinic, Technische Universität Dresden, Dresden, Germany; 15 Department of Otolaryngology - Head and Neck Surgery, Philippine General Hospital, University of the Philippines - Manila, Manila, Philippines; 16 Department of Psychology, University of Victoria, Victoria, BC Canada; 17 Department of Otorhinolaryngology – Head and Neck Surgery, Faculty of Medicine, Universiti Kebangsaan Malaysia, Kuala Lumpur, Malaysia; 18 Department of Otolaryngology, Cairo University, Cairo, Egypt; 19 Food, Flavour and Fragrance Lab, School of Psychological Sciences, Macquarie University, Sydney, Australia; 20 Department of Biomedical Sciences, University of Cagliari, SP 8 Cittadella Universitaria, Monserrato, Italy; 21 Department of Otolaryngology, Nippon Medical School, Tokyo, Japan; 22 Department of Surgery, Pritzker School of Medicine, the University of Chicago Biological Sciences Division, Chicago, USA; 23 Hospital Italiano de Buenos Aires, Buenos Aires, Argentina; 24 Department of Otolaryngology, Wroclaw Medical University, Wroclaw, Poland

**Keywords:** Olfaction, Neuroscience, Olfactory system, Olfactory sensitivity, Psychology, Ecology, Ecology, Neuroscience, Psychology, Psychology

## Abstract

It has been assumed that olfactory sensitivity is relatively consistent in different populations worldwide. Emerging, yet fragmented evidence lends credit to the hypothesis that olfactory sensitivity may be geographically diverse. To gain deeper insight regarding the interplay between geographical, demographic, and health factors in the context of olfactory sensitivity, we conducted a multicenter study comprising data from 1046 participants inhabiting 19 locations around the world. Our results revealed that location accounted for 17–20% of the variance in chemosensory sensitivity. Demographic and psychological factors related to working memory and depressive symptoms also contribute to explaining sensitivity to odors, accounting for 1.6–2.9% of the variance in chemosensory sensitivity. Thus, we conclude that inhabitants of different geographical regions may present different sensitivities to chemical stimuli. We discuss the factors that could potentially be included in future investigations to pinpoint even more precisely what factors determine differences in chemosensory sensitivity around the globe.

**Supplementary Information:**

The online version contains supplementary material available at 10.1038/s41598-026-38727-w.

## Introduction

Olfactory threshold is a reliable indicator of an individual’s sensitivity to odor concentrations in the environment. High sensitivity to odors serves as an alert system to environmental hazards such as leaking gas or spoiled food^1^. Despite the widespread belief that the human olfactory system is less sensitive than that of animals^2^, human olfactory sensitivity is on par with that of animals considered to rely heavily on their ability to detect odors^3^. Odor threshold reflects the function of the peripheral olfactory system, which is crucial for interaction with the physical environment and signal transmission to the central olfactory system. Unlike the suprathreshold ability to distinguish between odors (i.e. odor discrimination) and label them (i.e. odor identification)^4–9^ the olfactory threshold has been thought to be more independent of cultural background.

Most evidence of the cultural variability in chemosensory perception includes suprathreshold odor perception and self-reported measures of odor awareness and importance^5,10–19^. Recent cross-cultural studies^20^ showed that although there are some individual and cultural differences in the perception of odors, a significant part of the variability in odor pleasantness stems from the physicochemical properties of odorants^21^. At the same time, factors such as familiarity with smell and ecological context have a significant influence on cross-cultural differences in olfactory perception^22^. These results emphasize that olfactory experiences are shaped by universal biological constraints but modulated by culture and environment.

Emerging, yet fragmented evidence lends credit to the hypothesis that olfactory sensitivity may also be ethnically and geographically diverse. Research conducted in New York City found that African-Americans had higher olfactory sensitivity compared to Asians and Caucasians, while no difference was observed between Hispanic and non-Hispanic individuals^23^. Another study noted that members of the indigenous Tsimane tribe in Bolivia had a higher olfactory threshold compared to modern Germans^24^, and so were inhabitants of the Cook Islands, marginally exposed to ambient air pollution^25^. A study comparing Dutch and Japanese participants found no significant difference in sensitivity to six different odors^26^. Although ethnicity accounted for approximately 20% of the variation in olfactory perception, it has been suggested that factors such as previous upper respiratory infections, trauma, and environmental toxins play a more significant role than ethnicity^23^.

In 2020, Oleszkiewicz et al. attempted to examine global differences in human olfactory sensitivity, utilizing olfactory sensitivity data from 11 chemosensory laboratories across four continents^27^. Their findings attributed roughly 25% to 32% of the variation in olfactory/trigeminal and olfactory sensitivity (respectively) to geographic location, with gender and self-reported olfactory performance also predicting odor sensitivity. A second study (of the same paper) reaffirmed these results, indicating that changing a place of residence (i.e., moving from Asia to Europe) can impact the ability to detect odors^27^. The same research group observed that our sense of smell interplays with the olfactory landscape. Odors consciously detected in the environment can effectively stimulate the human olfactory system, potentially enhancing olfactory sensitivity^27^.

Understanding how environmental, demographic, and health factors shape olfactory sensitivity is relevant for the scientific public, medical practitioners, and policy makers. Understanding global diversity in olfactory sensitivity can inform public health policymakers of the need to adjust reference norms to the regional specificity and inspire more careful monitoring of the environmental exposure on population health. Results may turn out useful for product design to customize fragrant products to the regional variation in olfactory sensitivity.

To gain deeper insight regarding the interplay between environmental, demographic, and health factors in the context of olfactory sensitivity, we extended the previous global study on olfactory sensitivity^27^ in terms of the sample size, the number of locations, and the number of covariates considered. A network of specialized chemosensory laboratories conducted multicenter data collection. Care was taken to record individual characteristics hypothesized to explain the observed differences, such as demographics, health, and self-reported odor awareness. We intended to build a comprehensive, multilevel model explaining variability in human olfactory sensitivity across geographical regions, accounting for individual and regional factors. The current investigation focuses on the olfactory and olfactory/trigeminal sensitivity as the two chemosensory systems complement each other in aroma perception^1,28–31^ and interact with each other affecting olfactory perception^32,33^. Despite their relationship, little is known about the differential (or similar) cross-cultural variability of olfactory and olfactory/trigeminal sensitivity.

## Methods

### Ethical approval

The study was performed in accordance with the Declaration of Helsinki on Biomedical Studies Involving Human Subjects. Informed written consent was obtained from all participants. The study design and consent approach were approved by the Ethics Board at the Institute of Psychology (2021/RHYNA) and the Institutional Review Board of the University of Wroclaw (3/2021). The local institutional review board approvals were obtained where required.

### Study sample and eligibility criteria

The sample size was determined using G*Power software^34^. We anticipated small effects (*f*^2^ = 0.1) in this study, considering the inclusion of various relevant covariates and despite previous observations of large effect sizes^27^. We expected a small effect size because of the additional measures taken to increase random variance control (see Discussion for details). Exclusion criteria were pregnancy, smoking, regular exposure to chemical agents, neurodegenerative or metabolic diseases, infections on the day of measurement, diagnosed or self-reported olfactory dysfunction, and cognitive impairments.

The total sample of 1,046 participants (M_age_ = 32.2; SD_age_ = 12.3, 18–82 years) included 660 women (M_age_ = 32.4; SD_age_ = 12.3) and 382 men (M_age_ = 31.7; SD_age_ = 12.2) from 18 countries (Argentina, Australia, Brazil, Canada, China, Cuba, Egypt, Germany, India, Iran, Italy, Japan, Malaysia, Poland, the Philippines, Tunisia, Türkiye, United States of America). Two local samples were recruited in Canada (Trois-Rivières and Victoria), thus the study includes 19 locations. Table 1 summarizes the demographic characteristics of the population examined in each location.

**Table 1:** Descriptive statistics summary.

City	Country	Sample size	% Females	Age	
				M	SD
Buenos Aires	Argentina	65	54%	39.2	13.60
Cagliari	Italy	60	50%	29.2	10.83
Cairo	Egypt	66	50%	30.8	8.95
Chicago	USA	39	69%	32.8	18.37
Dresden	Germany	51	59%	26.4	5.57
Guangzhou	China	61	70%	32.4	12.60
Havana	Cuba	12	58%	44.3	14.94
Izmir	Türkiye	32	59%	33.4	15.44
Kuala Lumpur	Malaysia	65	78%	35.1	10.88
Londrina	Brazil	66	52%	30.5	13.86
Manila	The Philippines	60	62%	31.8	7.37
Pune	India	60	53%	31.3	10.60
Sydney	Australia	62	63%	25.2	10.60
Tehran	Iran	60	65%	34.3	10.92
Tokyo	Japan	65	60%	37.3	13.25
Trois-Rivières	Canada	22	86%	32.9	12.58
Tunis	Tunisia	65	74%	34.9	12.39
Victoria	Canada	70	76%	28.3	10.51
Wroclaw	Poland	65	69%	31.0	11.66
TOTAL		1046	63.1%	32.2	12.3

All subjects represented the urban population, because our partnering laboratories are located in the cities. In each subsample, the fraction of university students did not exceed 50%. The remaining participants were recruited from the general population and constituted a sample of convenience. An equal gender ratio was assumed at each location. To ensure that the recent change in residency did not alter the chemosensory perception, the participants included in this study resided in the area for more than 6 months. Our previous research has shown that this period appears sufficient to alter sensitivity to odors^27^. Data was collected between October 2022 and September 2024.

### Procedure

Local collaborating laboratories were recruited for the CROss-CUltural Study on Variability in Chemosensory Sensitivity (CROCUS) project using a professional network of colleagues who perform chemosensory research. All researchers contributing to this study have been previously trained or visited the Interdisciplinary Center for Smell and Taste at TU Dresden, Germany, and were all proficient in chemosensory testing and had extensive experience with the Sniffin’ Sticks. To ensure data quality, before data collection began, the researchers met twice for training and discussion on the study protocol. Additionally, the entire procedure was video recorded (including the procedure to calculate scores) and shared with each local research collaborator to ensure the coherence of the protocol across the locations. All chemosensory materials were purchased in Germany and sent to the local collaborators via courier.

At each location, all participants underwent individual testing sessions conducted in well-ventilated and properly lit rooms. Breaks were offered to the participants at any time in case of signaled fatigue. Questionnaires and methods with documented psychometric properties were employed to assess dependent and control variables. In cases where native language adaptations of scales were unavailable, bilateral translations were conducted. The duration of each session was estimated to be 60 min.

### Dependent variables

#### Olfactory sensitivity

For the measurement of olfactory sensitivity, we used the Sniffin’ Sticks threshold test^7^. The test employs a triple-forced choice paradigm, where participants must differentiate the odorous target pen from two blanks filled with propylene glycol solvent. The odorous pen was filled with a mixture of ten odorants mixed in equal volumes (see Table 2 for details). The highest concentration was 0.25%, and further dilutions were prepared in a 1:3 fashion, resulting in a total of 8 concentrations. The use of an 8-step version of the olfactory sensitivity test (instead of the full 16-step) served to shorten the examination procedure and avoid errors resulting from the fatigue of the subjects. Both the odor and blanks are presented about 2 cm in front of the participant’s nostrils for 3 s. Starting with the lowest odor concentration, a staircase paradigm adjusts the concentration based on the participant’s responses. Two correct or one incorrect answer led to a decrease or increase in concentration, respectively, termed a turning point. The test completes seven turning points, with the final score being an average value of the last four. To ensure reliability, the test was performed twice with each participant with a 15-min break^35^.

**Table 2:** Odorants constituting the threshold test mixture.

Name	Manufacturer	Product code
p-Cymene	Sigma-Aldrich	C121452
4-(4-Hydroxyphenyl)−2-butanone	Sigma-Aldrich	178,519
Cinnamaldehyde	Sigma-Aldrich	C80687
Sandalwood oil	Sigma-Aldrich	W300500
Octanal	Sigma-Aldrich	O5608
Heptanal	Sigma-Aldrich	W254002
Benzylalcohol	Sigma-Aldrich	402,834
1-Phenylethyl acetate	Sigma-Aldrich	8.43785
Acetophenone	Sigma-Aldrich	8.00028
Propylene glycol	Sigma-Aldrich	424,927-1L

#### Olfactory/trigeminal sensitivity

An analogous method was used to measure olfactory/trigeminal sensitivity; however, Sniffin’ Sticks were filled with dilutions of Eucalyptol (highest concentration 0.03125%). Olfactory/trigeminal sensitivity was measured once.

### Predictors of olfactory and olfactory/trigeminal sensitivity

#### Olfactory-related health

Olfactory-related health conditions were checked using a standardized medical form used in chemosensory studies^36^ regarding general health and past medical conditions. The interview included all exclusion criteria established for this study, such as nasal polyps or a history of head trauma. The participants assessed their sense of smell on a six-point Likert-type scale (ranging from 1—very good to 6—no smell perception) and nasal breathing by determining the patency of each nostril. In addition, questions about COVID-19 were added to the questionnaire to document potential infections, symptoms (such as loss of smell), and treatment.

#### Peak nasal inspiratory flow (PNIF)

Peak nasal inspiratory flow (PNIF) is a rapid and easy-to-use objective measure that directly measures nasal airflow during maximal inspiration^37^. We measured PNIF to control for nasal airway obstructions in our participants. We asked each participant to inhale air through the nose at maximum speed and effort. The measurement was taken in a standing position three times, and the maximum score of the three trials was included in the analyses.

#### Depression

Depressive symptoms are associated with reduced olfactory abilities^38^. We used PHQ-9 Questionnaire, a self-administered tool for assessment of depressive symptoms. PHQ-9 includes 9 items which focus on DSM- IV criteria, such as anhedonia (reduced ability to experience pleasure), weight (less or more appetite), sleep (more or less than usual sleep), psychomotor changes, fatigue (loss of energy), worthlessness (feeling of being unworthy), concentration (lack of concentration), suicidal thoughts and behaviors^39,40^.

#### Digit span

The Digit Span test was used to assess attention and auditory short-term memory^41^. In this task, the participants listened to a series of digits presented verbally and were asked to repeat them in the same order. The test began with a sequence of three digits, the length of which increased by one digit on each successive trial up to 11 digits. The test ended if the participant made an error on two successive trials of the same length. The total score was determined by the number of sequences correctly recalled, with possible scores ranging from 0 to 9 points.

#### Verbal fluency

Verbal fluency was checked by using the Controlled Oral Word Association Test^42^. COWAT is a verbal fluency test that measures the spontaneous production of words belonging to the same category or beginning with some designated letter. It is thought to measure linguistic abilities and executive functions^43^. In our study, the participants were asked to name as many animals as they could within 30 s.

#### Individual significance of olfaction

The Individual Significance of Olfaction scale was employed to evaluate the personal importance of the sense of smell^44^. This scale comprises 18 items divided into three subscales: ‘Association’, ‘Application’, and ‘Consequence’. Additionally, two items from the ‘Aggravation’ subscale were included to detect any tendency of the participants to overestimate the significance of their sense of smell. The ‘Association’ subscale assesses emotions, memories, and evaluations triggered by olfactory experiences. The ‘Application’ subscale measures how individuals use their sense of smell in everyday life. The ‘Consequence’ subscale examines the role of olfaction in daily decisions. The reliability of the questionnaire is reported to be α = 0.77. The results from this scale were analyzed separately for each subscale to provide a comprehensive understanding of the significance of olfaction in daily life. The overall score is the average value of all items and ranges from 0 to 3 points.

#### Age

Olfactory sensitivity changes with age. A significant development of olfactory function is observed during the first 20 years of life^45,46^, whereas a decline in olfactory sensitivity occurs after 60 years of age^45,47,48^. Variations in age demographics and life expectancy rates across populations^49^ may influence olfactory sensitivity in different countries.

#### Gender

Differences in olfactory sensitivity are observed between the genders. Women exhibit slightly higher olfactory performance^50–52^ and value odors more than men^44^.

### Statistical analyses

Data were analyzed using RStudio software Version 2024.12.0 + 467 (packages “lme4”, “lmerTest”, “MuMin”, “tidyverse”, “ggplot2”, “ggpubr”). First, we assessed the reliability of the olfactory threshold test with Pearson’s r correlation calculated for the first and the second measurement of olfactory sensitivity. To reflect the nested structure of the data set, separate within- and between-location variance, and to allow generalization beyond the sampled locations, we used multilevel linear modeling (MLM) with random intercepts and random slopes. Gender and health status were included as fixed factors (nominal variables). Age, PNIF, depressive symptoms, individual significance of olfaction, digit span, and verbal fluency were included as fixed covariates (scale variables). Location was treated as a random factor. Two models were tested separately for olfactory and olfactory/trigeminal thresholds. Conditional R^2^ was used to quantify the proportion of variance explained by the fixed and random effects (total variance explained by the model). Marginal R^2^ indicated the proportion of variance explained by the fixed factors and fixed covariates (Gender, Age, Health status, PNIF, Depressive Symptoms, Individual Significance of Olfaction, Digit Span, and Verbal Fluency). Thus, the difference between Marginal R^2^ and Conditional R^2^ indicated the proportion of variance explained by location^20,27,53^.

## Results

### Reliability of olfactory threshold testing

**Fig. 1:**
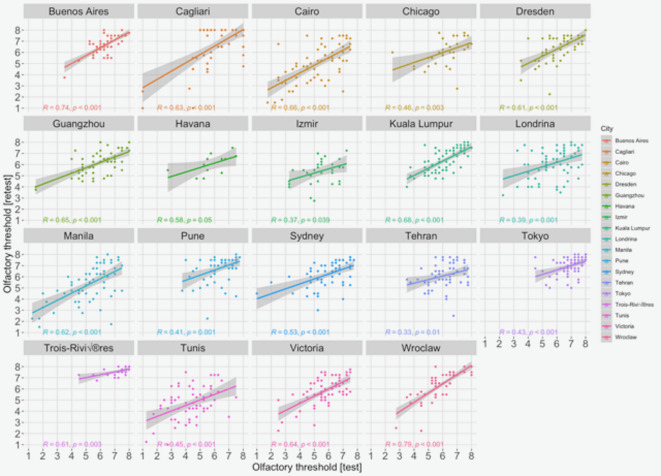
Test–retest reliability was adequate and comparable across the locations.

The overall correlation coefficient for olfactory threshold first and second measurement was *r*_1044_ = 0.65, which was similar to the test–retest reliability for odor threshold tests based on odor mixtures (r = 0.56 for six and r = 0.56 for ten odors)^54,55^. Figure 1 shows correlations between scores in olfactory threshold for the first and second attempts across the tested locations. In all the locations, these two measurements were positively and robustly correlated; therefore, we assumed the reliability of the test across the locations. Further analyses were based on the score from the first olfactory threshold test attempt.

### Descriptive statistics across the locations

**Fig. 2:**
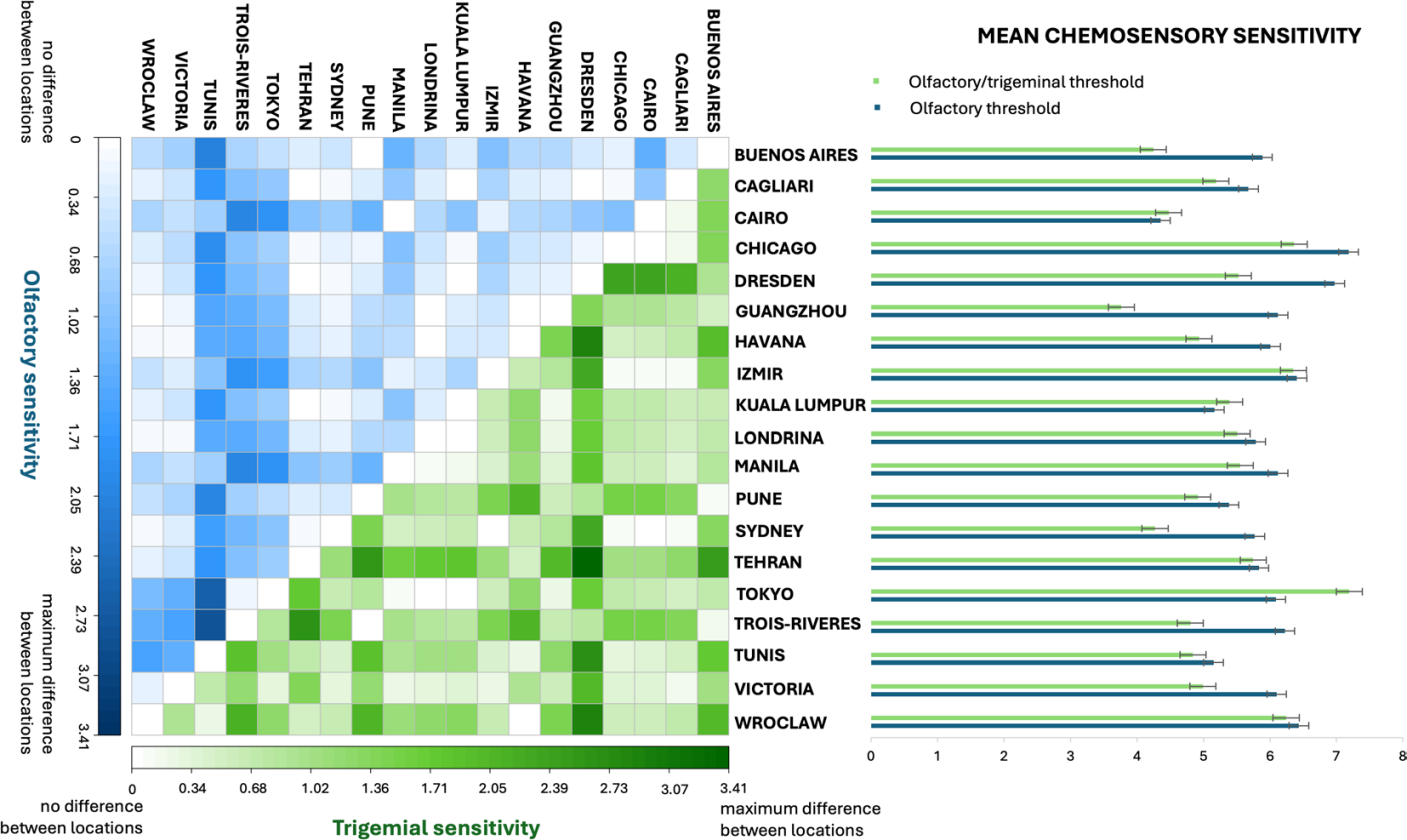
Heatmap (left panel) presenting differences between mean scores obtained across locations for olfactory sensitivity (in blue) and trigeminal sensitivity (in green). Bar graph (right panel) showing mean scores in olfactory (in blue) and olfactory/trigeminal (in green) tests across the study locations. Heatmap: Y-axis represents a gradient of blue color to mark the pair-wise distances between the locations in olfactory sensitivity. The X-axis represents the gradient of green color to mark the pair-wise distances between the locations in olfactory/trigeminal sensitivity. Each tile represents a single pairwise comparison. Darker tiles indicate larger differences between the locations. Bar chart: Y-axis maps the locations. The X-axis represents the possible score range in the olfactory and olfactory/trigeminal threshold test. Bars depict mean scores in both threshold tests for each location. Error bars denote the standard error of the mean. Lower scores indicate lower sensitivity to odors (i.e. higher thresholds) and higher scores indicate higher sensitivity to odors (i.e. lower thresholds).

Table S1 in the Supplementary Materials presents the descriptive statistics for the test and retest olfactory threshold test scores across the locations. Pairwise mean differences in olfactory threshold and trigeminal threshold across the locations are summarized in the heatmap in the left panel of Fig. 2. In the right panel of Fig. 2, bar graphs display mean sensitivity scores for olfactory and trigeminal modalities across locations.

### Geographical variability in olfactory sensitivity

The linear mixed model accounted for 19.5% of the variability in olfactory thresholds (as indicated by the Conditional R^2^, i.e. the variance explained by fixed and random effects), with 17.9% of this variability being explained by the location (as indicated by the difference between Conditional R^2^ and Marginal R^2^, the latter accounting for the variance explained only by fixed factors) (see Table 3). The remaining 1.6% of the variance (R^2^ Marginal) was attributed to the individual characteristics of the participants. Olfactory sensitivity decreased with age (B = −0.01; *p* = 0.02); and depressive symptoms, wherein higher levels of depressive symptoms were associated with reduced ability to detect odors (B = −0.02; *p* = 0.01). There were no other significant predictors within the tested model, although Gender, PNIF bordered significance, pointing to the elevated olfactory sensitivity in women and individuals with increased nasal airflow.

**Table 3:** Model predicting olfactory threshold based on individual-level predictors and location.

**Model A**	Estimate	Std. error	df	t	Sig	R^2^_partial_
Intercept	6.43	.30	354.6	20.3	<.001	
Age	-.01	<.01	1034	−2.37	.02	.004
Gender (ref. = men)	.16	.09	1030	1.87	.06	.003
PNIF	.002	<.01	1036	1.95	.06	.004
Health status (ref. = healthy)	-.084	.08	1038	−1.01	.31	<.001
Digit span	-.04	.03	954	−1.54	.12	.002
Verbal fluency	-.01	.01	1035	−1.29	.20	<.001
Depression	-.02	.01	1040	−2.50	.01	.006
Individual significance of olfaction	.02	.10	1040	.26	.80	.0001
Model fit						
AIC	3447.69					
BIC	3502.13					
−2LL	−1712.85					
Variance explained	19.5%					
R^2^ marginal (fixed factors)	.016					
R^2^ conditional (fixed + random factors)	.195					

ref = reference category, PNIF = Peak nasal inspiratory flow, AIC = Akaike information criterion, BIC = Bayesian information criterion, −2LL = −2 log likelihood, R^2^—variance.

### Geographical variability in olfactory/trigeminal sensitivity

The model predicting olfactory/trigeminal threshold accounts for 19.7% of the variability in olfactory/trigeminal thresholds (as indicated by the Conditional R^2^ i.e. the variance explained by fixed and random effects) with 16.8% attributed to the location (as indicated by the difference between Conditional R^2^ and Marginal R^2^, the latter accounting for the variance explained only by fixed factors) (see Table 4). The remaining 2.9% of the variance (R^2^ Marginal) was linked to individual participant characteristics. Women exhibited higher olfactory/trigeminal threshold scores compared to men (B = 0.43; *p* = < 0.001); olfactory**/**trigeminal sensitivity increased in the absence of comorbid health issues (B = −0.27; *p* = 0.02); higher scores on the digit span test were associated with an increase in trigeminal olfactory sensitivity thresholds (B = 0.11; *p* = 0.04). No additional significant predictors were identified within the tested model.

**Table 4:** Estimated for the model predicting olfactory/trigeminal threshold based on individual-level predictors and location.

**Model B**	Estimate	Std. error	df	t	Sig	R^2^_partial_
Intercept	4.99	.45	370.4	11.14	<.001	
Age	-.01	.01	1034	−1.25	.21	.001
Gender (ref. = men)	.43	.12	1029	3.50	<.001	.011
PNIF	.002	.002	1035	1.14	.25	.002
Health status (ref. = healthy)	-.27	.12	1037	−2.26	.02	.006
Digit span	.11	.04	940.5	2.66	.01	.011
Verbal fluency	-.01	.01	1032	-.69	.49	.001
Depression	-.004	.01	1039	-.33	.74	<.001
Individual significance of olfaction	-.16	.13	1039	−1.20	.23	.001
Model fit						
AIC	4177.76					
BIC	4232.17					
−2LL	−2077.88					
Variance explained	19.7%					
R^2^ marginal (fixed factors)	.029					
R^2^ conditional (fixed + random factors)	.197					

Note. ref = reference category, PNIF – Peak nasal inspiratory flow,AIC = Akaike information criterion, BIC = Bayesian information criterion, −2LL = −2 log likelihood, R^2^—variance.

**Fig. 3:**
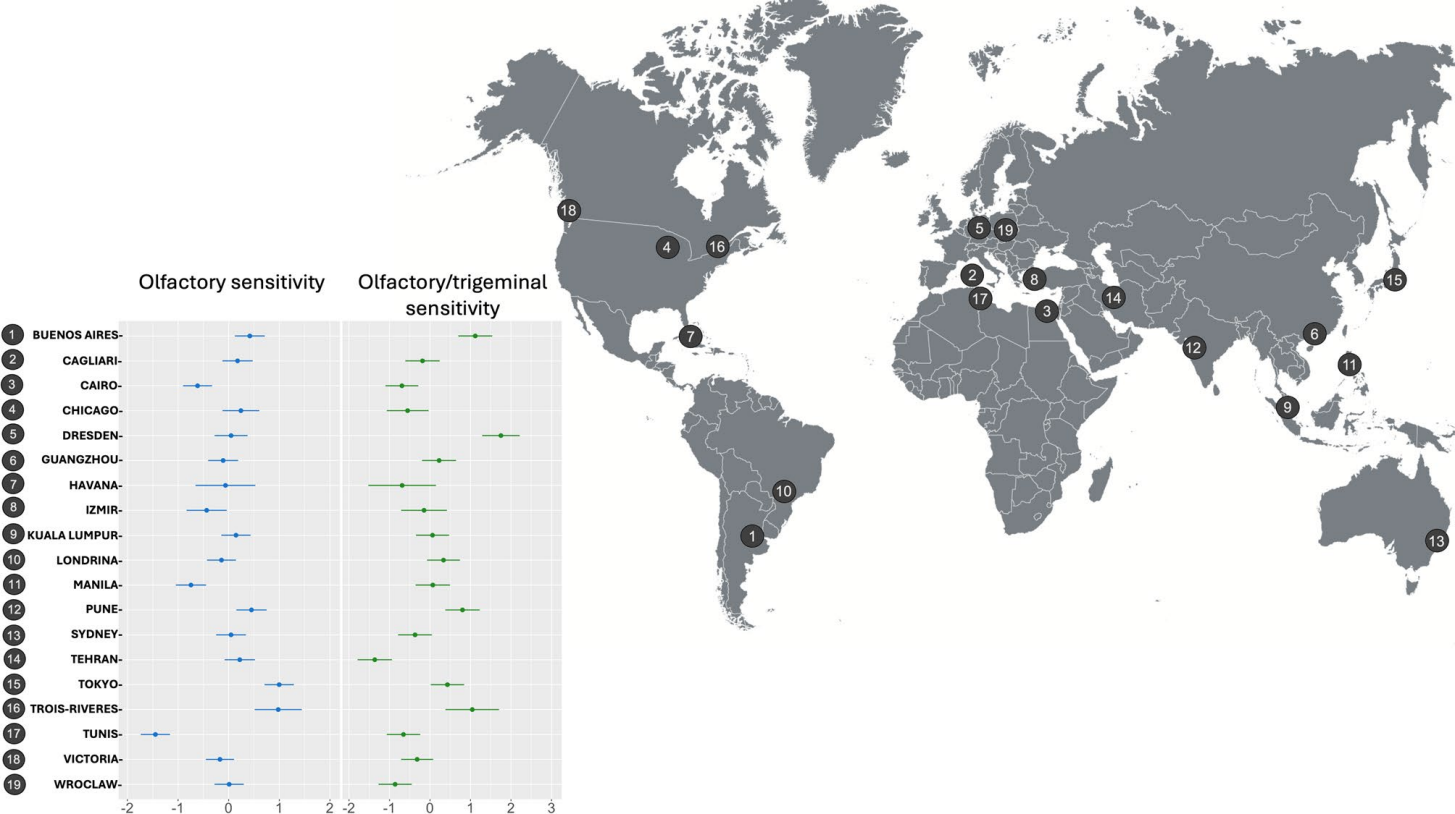
Forest plots mapping the spread of locations (random effects) around the covariate-adjusted grand mean (marked with dashed black line) for olfactory (left panel, blue color) and olfactory/trigeminal (right panel, green color) sensitivity. Error bars denote 95% confidence intervals. The world map (right panel) summarizes testing locations. Figure 3 was created by Aleksandra Reichert with Canva Software (version: Canva 2025; link: https://www.canva.com/).

Forest plots illustrating city-level effects around the covariate-adjusted grand mean for olfactory and olfactory/trigeminal sensitivity are shown in Fig. 3.

## Discussion

The current study examined global olfactory sensitivity in 19 locations, taking into account individual factors and geographical location. Our results revealed that location accounted for 16.8–17.9% of the variance in chemosensory sensitivity.

### Current findings in the context of previous work

Despite a larger sample size and broader geographic coverage, several additional covariates considered, more rigorous protocol standardization, and a modelling approach, the obtained results are consistent with the initial study our network has published^27^. Previously, we observed a slightly higher variance attributed to the location than in the current study (32% vs 20% in olfactory sensitivity and 25% vs 17% in olfactory/trigeminal sensitivity). The lower variance between the study sites, despite the extended collaboration network, can be attributed to a more rigorous protocol, corroborated by video conferences to discuss nuances of the study protocol and the video recording of the protocol. Furthermore, in the current study, we used an odor mixture to quantify olfactory sensitivity^54,55^ while in the pilot study^27^ we used the Sniffin’ Sticks test based on the single-molecule PEA odor. We controlled some of the variance by testing olfactory sensitivity twice during the testing sessions and found robust test–retest correlations in all regional subsamples. Thus, we conclude that the diminished variance in thresholds between the locations results from minimizing the experimenter’s effect and additional efforts to standardize the protocol. Despite our best efforts, we acknowledge that a portion of the variance attributed in this study to the location may still result from subtle differences in protocol execution between the testing sites. An ideal solution to address this problem would be to collect data by the same person across various locations, but such an approach would entail language barriers, confounds attributable to the cultural differences between the experimenter and the participants, and potentially language issues. Although we find some locations to have strong and other locations to have moderate correlations between the olfactory threshold test and retest, it must also be acknowledged that its reliability should be assessed somewhat more liberally than the classic psychometric benchmark^56,57^.

Selected individual-level factors, namely gender, age, health status, PNIF, depression, individual significance of olfaction, digit span, and verbal fluency, accounted for a smaller proportion of variance than the location (for olfactory threshold 1.6% and for olfactory/trigeminal threshold 2.9% of variance). We corroborated previously reported small gender differences in olfactory/trigeminal sensitivity in favor of women^51,52,58^ and demonstrated a well-established decline in olfactory sensitivity with age^47,59^. Beyond these demographical characteristics of our subjects, we found that working memory, depressive symptoms, and general health status to determine chemosensory acuity. Specifically, individuals with better memory for digits presented lower olfactory/trigeminal thresholds. Working memory is a marker of cognitive functions. It is known to decline with aging, development of mild cognitive impairment, and neurodegenerative diseases^60–62^, similar to chemosensory sensitivity^63–65^. The nature of both tests – the digit span and olfactory/trigeminal threshold task, both engage working memory. The participant was either memorizing digits or chemosensory sensations in the sequence of the presented pens. Because our models accounted for age and at least half of our sample recruits from the general population, the digit span is likely a marker of cognitive abilities that may have been compromised in some of the participants, along with the performance in the olfactory/trigeminal threshold task^66^. This, however, cannot be verified in more depth as we did not collect any data that would explain the potential cause of diminished working memory. Given that olfactory threshold is less dependent on executive functions than olfactory discrimination and identification^67^, we treat this finding with caution.

More severe depressive symptoms were associated with lower olfactory sensitivity. The body of evidence links depression with compromised olfactory abilities. Evidence gathered for this study is correlational. We cannot draw any causal statements of whether the observed relationship reflects the overall anhedonia and reluctance to participate in chemosensory tasks in the participants with elevated depressive symptoms, or whether their olfactory function reflected structural and functional changes in the olfactory system^38,68–73^.

In line with previous reports, we did not observe nasal airflow to be associated with olfactory sensitivity^27^. Our subjects were recruited from the academic/general populations and considered themselves healthy enough to participate in the olfactory study. In contrast, patients with chronic rhinitis complain about nasal airflow and present decreased olfactory sensitivity; however, their objective nasal airflow appears not to correlate with olfactory sensitivity. Thus, in a clinical sample, factors such as inflammatory states, trigeminal dysfunction, and anatomical features may relate to decreased olfactory perception rather than PNIF^74^.

The presence of health issues potentially related to olfactory dysfunction, such as head injuries, allergic rhinitis, bronchial asthma, nasal polyps, or frequent respiratory infections, was negatively associated with olfactory/trigeminal sensitivity. We did not observe the olfactory threshold to be related to the health problems. Potentially, our test was not sensitive enough to differentiate people varying in health status^23,75,76^. In the future, the use of a full 16-step olfactory threshold test (instead of an 8-step) could yield more consistent results.

### Limitations

The estimated location effects likely conflate several factors, including length of residence, ethnicity and ancestry, socio-economic and cultural practices, all potentially mediating chemosensory experience and vigilance to odors. Our study warrants a potentially interesting avenue of research focusing on cultural embeddedness and chemosensory sensitivity. Although some facts have been established, the dominant conclusions are limited to the mere fact of cultural differences in chemosensory sensitivity^24,27^, and more detailed investigations are needed to increase our understanding of the chemosensory experience and performance in different cultures. Efforts to recruit locations across cultural regions (e.g., North America, Europe, Asia) or climatic zones (e.g. Equatorial, Tropical, Temperate, Polar) or to incorporate geographic or cultural distance measures (but see Sorokowska et al. 2018) would further clarify the role of location in shaping chemosensory sensitivity.

Certain factors remained beyond our control, but their potential contribution to a better understanding of the factors that shape regional diversity in olfactory sensitivity deserves discussion. The total sample is likely to be rather educated (half of the sample is academic, the other, non-academic half is a sample of convenience), and in relatively good health, as no clinical groups were targeted. To participate in an olfactory study, a certain interest in odors is also likely to occur, leaving out of the sample individuals with little interest in odors. Although we conducted a standard medical interview regarding olfactory function, our models were based on self-assessment of health status. We did not take into account objective health indicators or clinical data that could have increased the accuracy of our results. Nevertheless, this interview allowed us to exclude individuals with evident olfactory disorders and other acute health problems, which increases the validity of the study sample. In sum, our local samples should not be regarded as representative to the local population.

Our study is missing control over genetic factors likely explaining part of the variability in olfactory thresholds. Although our understanding of how genes determine our perception of odors is far from complete, preliminary evidence appears to show evidence shows that genes can shape olfactory acuity. Behavioral research in twins demonstrated that olfactory sensitivity to specific odors is not heritable^76^, but the expression of certain olfactory receptors can result from specific stimulation^77,78^. Polymorphisms in OR11H7P, TRPA1 or SCN9A genes may partially shape sensitivity towards particular odorants^79^. Individuals with functional alleles of these genes typically exhibit heightened olfactory sensitivity. In contrast, those with non-functional alleles may show reduced sensitivity or anosmia for specific odors^80^. Regrettably, our study does not encompass the genetic profiles of the participants.

The sample can also be assessed as relatively young. Age span was not a significant predictor of sensitivity to eucalyptus. The trigeminal system is less vulnerable to age-related functional decline than the olfactory system; therefore, in our sample, a potential decrease in olfactory/trigeminal sensitivity with age may not have been detected^81^. With a more age-diverse sample size, we could potentially explain more variance with individual-level factors.

Another uncontrolled factor that could potentially explain more variability in chemosensory sensitivity may be the ethnicity of participants. Keller and colleagues^23^ established that the perception of odors varies significantly across demographic groups, also as a function of ethnicity. According to a meta-analysis^82^ ethnicity may account for up to 20% of the variability in olfactory perception, but its impact appears to be smaller than that of environmental factors. It remains unclear to what proportion of unexplained variance ethnicity accounted for in our study focusing on olfactory sensitivity, and to what extent this variance is unique from location. Future studies would benefit from combining these two factors. The participants in our study resided in the investigated location for at least 6 months. This requirement was set to assure sensory adaptation^27^, however, six months may still be relatively short to treat the participants as representative residents of a city or cultural region in a broader cultural and lifestyle sense. This is a potential limitation that could be mitigated in the future by additionally controlling for the length of residence and the assessment of cultural assimilation.

### Future directions

All local subsamples in this study were recruited among urban dwellers. Studies have shown significant differences in olfactory sensitivity between urban and rural populations^83^ and Indigenous and modernized societies^25,84^. In this context, our study sample may appear homogenous, yet olfactory sensitivity can also change with air pollution specific to different urban regions^85^. Modeling air pollution composition together with atmospheric conditions such as air pressure, humidity, temperature, and insolation is extremely complex. It should rather be systematically examined with its impact on human chemosensory abilities in controlled lab settings than in natural settings. Evidence so far suggests rather negligible effects of atmospheric conditions on human olfactory sensitivity^86^. A major challenge is controlling for the ambient air pollution exposure before the event of chemosensory threshold testing, to account for toxic agents causing potential damage to the peripheral olfactory system.

Most people spend most of their time indoors, where pollution levels can exceed outdoor levels due to significant indoor emissions^87,88^. Surprisingly, the knowledge about the harmful effects of indoor air pollution on human health is scarce. Indoor air pollution has been shown to have adverse effects on the respiratory and neurological systems, exacerbate asthma and allergy symptoms, and cause throat and nasal irritation^89^. It can therefore be assumed that exposure to such factors may also impair the sense of smell, including olfactory sensitivity, but direct evidence on this relationship is missing. An intriguing line of research would be measuring exposure to ambient air pollution with personal devices monitoring indoor and outdoor air quality in the proximity of the subject.

### Practical implications

Results of our investigation have applicational potential. The functional olfaction is relevant for nutrition, environmental safety, and social well-being^1,90^. Thus, more careful monitoring of the environmental exposures, and screening of olfaction-related health in the regions where individuals exhibit compromised chemosensory sensitivity should be of public health policy makers’ interest. Based on these results, medical professionals may want to revisit reference norms in the future to adjust them to the population level of olfactory sensitivity. Finally, our findings may turn out to be useful for the product designers, who can adjust the intensity of smell in their products to the regional variability in olfactory and olfactory/trigeminal sensitivity.

## Conclusion

With a broader and more rigorous approach, our study replicated and extended the preliminary findings suggesting that a unique portion of the variance in olfactory sensitivity can be attributed to one’s geographical location. Additionally helpful in predicting olfactory acuity are demographic and psychological factors related to working memory and depressive symptoms. People inhabiting different regions may have different sensitivities to chemical stimuli due to varying exposures to atmospheric conditions and different chemosensory experiences in daily life.

## Supplementary Information

Supplementary Information.

## Data Availability

All data associated with this manuscript are publicly available: https://osf.io/m95z4/
