# Supplementary material for: Human olfactory sensitivity varies across geographical locations
Source: Sci Rep. 2026 Feb 18;16:9713. doi: 10.1038/s41598-026-38727-w (PMC13013636; doi:10.1038/s41598-026-38727-w)
Supplement: Supplementary file 1 — Supplementary Information. [file 41598_2026_38727_MOESM1_ESM.pdf]

Supplementary materials
"Human olfactory sensitivity varies across geographical locations"

Table S1: Descriptive statistics for the test and retest olfactory threshold test scores across the locations.

			95% Confidence Interval of the Mean					
	**City**	**Mean**	**Lower**	**Upper**	**Median**	**SD**	**Min**	**Max**
Odor threshold test score – first measut	Buenos Aires	6.43	6.23	6.64	6.50	0.835	3.50	8.00
	Cagliari	6.10	5.70	6.50	5.88	1.547	1.00	8.00
	Cairo	5.15	4.76	5.54	5.25	1.595	1.75	7.50
	Chicago	6.22	5.84	6.61	6.25	1.189	2.50	8.00
	Dresden	6.09	5.77	6.41	6.25	1.139	3.50	8.00
	Guangzhou	5.83	5.49	6.18	6.00	1.340	1.50	8.00
	Havana	5.77	4.96	6.58	5.63	1.281	2.75	7.50
	Izmir	5.38	4.98	5.78	5.38	1.103	3.25	7.25
	Kuala Lumpur	6.12	5.85	6.39	6.25	1.103	3.50	8.00
	Londrina	5.78	5.47	6.10	6.00	1.266	2.25	7.75
	Manila	5.16	4.76	5.57	5.50	1.566	1.25	7.50
	Pune	6.40	6.15	6.66	6.50	0.996	3.75	7.75
	Sydney	6.01	5.66	6.36	6.38	1.371	1.00	7.75
	Teheran	6.12	5.83	6.41	6.25	1.116	3.50	7.75
	Tokyo	6.97	6.76	7.18	7.25	0.848	4.50	8.00
	Trois-Rivières	7.18	6.76	7.60	7.50	0.942	4.50	8.00
	Tunis	4.35	4.03	4.68	4.50	1.321	1.25	7.50
	Victoria	5.67	5.40	5.95	5.63	1.168	2.50	7.50
	Wroclaw	5.88	5.60	6.17	5.50	1.156	2.75	8.00
Odor threshold score (2)	Buenos Aires	6.72	6.52	6.91	6.75	0.787	3.75	8.00
	Cagliari	6.63	6.16	7.11	7.75	1.840	1.00	8.00
	Cairo	4.94	4.54	5.35	4.88	1.647	1.50	7.50
	Chicago	6.08	5.71	6.46	6.00	1.149	2.75	7.75
	Dresden	6.35	6.01	6.68	6.75	1.196	2.25	8.00
	Guangzhou	6.11	5.86	6.37	6.25	1.007	3.75	8.00
	Havana	6.04	5.48	6.61	6.25	0.891	4.75	7.50
	Izmir	5.38	4.98	5.79	5.50	1.129	2.75	7.25
	Kuala Lumpur	6.39	6.13	6.64	6.30	1.041	4.00	8.00
	Londrina	6.11	5.79	6.43	6.25	1.288	3.25	8.00
	Manila	5.28	4.86	5.71	5.50	1.641	1.50	8.00
	Pune	6.78	6.49	7.07	7.25	1.110	2.25	8.00
	Sydney	6.24	5.95	6.54	6.50	1.147	3.00	7.75
	Teheran	6.10	5.82	6.39	6.13	1.088	2.50	7.75
	Tokyo	6.99	6.80	7.19	7.25	0.789	5.00	8.00

	Trois-Rivières	7.57	7.39	7.74	7.63	0.395	6.75	8.00
	Tunis	4.70	4.34	5.05	4.75	1.437	1.00	7.50
	Victoria	5.79	5.51	6.07	5.75	1.167	2.25	7.75
	Wroclaw	6.36	6.06	6.66	6.50	1.205	2.25	8.00
Trigeminal/olfactory threshold test score	Buenos Aires	6.24	5.95	6.53	6.50	1.163	3.25	8.00
	Cagliari	4.99	4.27	5.71	5.50	2.779	1.00	8.00
	Cairo	4.84	4.44	5.25	4.88	1.648	1.75	7.50
	Chicago	4.80	4.28	5.32	5.50	1.608	1.00	7.25
	Dresden	7.19	6.76	7.63	7.75	1.548	1.00	8.00
	Guangzhou	5.75	5.30	6.19	6.25	1.729	1.00	8.00
	Havana	4.27	3.59	4.96	4.00	1.079	2.75	5.75
	Izmir	4.91	4.32	5.51	5.50	1.654	1.50	7.00
	Kuala Lumpur	5.55	5.14	5.96	5.25	1.648	1.75	8.00
	Londrina	5.51	5.10	5.92	5.88	1.658	2.00	8.00
	Manila	5.39	4.99	5.79	5.50	1.556	1.25	8.00
	Pune	6.35	5.84	6.86	7.00	1.959	0.00	8.00
	Sydney	4.93	4.48	5.38	5.25	1.770	1.00	7.75
	Teheran	3.76	3.37	4.16	4.13	1.521	1.00	7.25
	Tokyo	5.52	5.04	6.01	6.25	1.964	1.00	8.00
	Trois-Rivières	6.36	5.68	7.04	6.88	1.533	3.00	8.00
	Tunis	4.48	3.97	4.98	4.50	2.022	1.00	8.00
	Victoria	5.19	4.75	5.62	5.75	1.840	1.00	8.00
	Wroclaw	4.25	3.88	4.62	4.50	1.489	1.00	7.25
